# Complex Environmental Geomagnetic Matching-Assisted Navigation Algorithm Based on Improved Extreme Learning Machine

**DOI:** 10.3390/s25144310

**Published:** 2025-07-10

**Authors:** Jian Huang, Zhe Hu, Wenjun Yi

**Affiliations:** National Key Laboratory of Transient Physics, Nanjing University of Science and Technology, Nanjing 210094, Chinahz@njust.edu.cn (Z.H.)

**Keywords:** geomagnetic matching assisted navigation, magnetic sensor, Northern Goshawk Optimization algorithm, Extreme Learning Machine

## Abstract

In complex environments where satellite signals may be interfered with, it is difficult to achieve precise positioning of high-speed aerial vehicles solely through the inertial navigation system. To overcome this challenge, this paper proposes an NGO-ELM geomagnetic matching-assisted navigation algorithm, in which the Northern Goshawk Optimization (NGO) algorithm is used to optimize the initial weights and biases of the Extreme Learning Machine (ELM). To enhance the matching performance of the NGO-ELM algorithm, three improvements are proposed to the NGO algorithm. The effectiveness of these improvements is validated using the CEC2005 benchmark function suite. Additionally, the IGRF-13 model is utilized to generate a geomagnetic matching dataset, followed by comparative testing of five geomagnetic matching models: INGO-ELM, NGO-ELM, ELM, INGO-XGBoost, and INGO-BP. The simulation results show that after the airborne equipment acquires the geomagnetic data, it only takes 0.27 µs to obtain the latitude, longitude, and altitude of the aerial vehicle through the INGO-ELM model. After unit conversion, the average absolute errors are approximately 6.38 m, 6.43 m, and 0.0137 m, respectively, which significantly outperform the results of four other models. Furthermore, when noise is introduced into the test set inputs, the positioning error of the INGO-ELM model remains within the same order of magnitude as those before the noise was added, indicating that the model exhibits excellent robustness. It has been verified that the geomagnetic matching-assisted navigation algorithm proposed in this paper can achieve real-time, accurate, and stable positioning, even in the presence of observational errors from the magnetic sensor.

## 1. Introduction

In the navigation system of an aerial vehicle, it is crucial to obtain accurate and real-time position information. Currently, aerial vehicles primarily rely on a combined navigation system that integrates an Inertial Navigation System (INS) and Global Positioning System (GPS) for positioning [1]. INS tends to accumulate system errors over time, which hinders its ability to meet the demands for high-precision positioning. Therefore, it needs to be combined with GPS for navigation. However, GPS may experience signal loss or even fail to provide positioning in extreme environments [2,3,4,5]. Geomagnetic navigation technology, which utilizes the spatial variation of the geomagnetic field, offers advantages such as all-terrain applicability, all-weather capability, strong anti-jamming performance, and no error accumulation. Consequently, geomagnetic matching technology can provide stable and precise positioning for aerial vehicles. By integrating geomagnetic navigation with inertial navigation, high-precision autonomous navigation for aerial vehicles can be achieved [6].

The key technologies of geomagnetic navigation can be categorized into geomagnetic filtering and geomagnetic matching [7]. Geomagnetic filtering technology associates the measured magnetic field data with the INS data, and corrects the position output of the INS through filtering. Common geomagnetic filtering techniques include Kalman filtering, particle filtering, etc. Among the geomagnetic navigation systems based on Kalman filtering, the most representative one is the Sandia Inertial Terrain Aided Navigation (SITAN) [8,9]. The SITAN algorithm continuously processes the INS data through Kalman filtering and estimates the position information by combining terrain elevation. However, when the terrain information is linearized or the initial position accuracy is poor, the navigation accuracy deteriorates, resulting in poor robustness of the SITAN algorithm [10,11]. Therefore, the theory of nonlinear filtering has attracted increasing attention from relevant scholars. Stepanov and Toropov [12] formulated the map-aided navigation problem within the framework of Bayesian nonlinear filtering theory, providing a rigorous theoretical foundation for the design of corresponding nonlinear filtering algorithms. Particle filtering, a nonlinear filtering method leveraging the Monte Carlo approach, enables parameter estimation in nonlinear and non-Gaussian environments [13]. It approximates the posterior probability distribution through a large number of particles, leading to a significant increase in computational cost and insufficient real-time performance. Furthermore, particle filtering suffers from the issues of sample degradation and sample impoverishment [14,15]. In comparison to geomagnetic filtering, geomagnetic matching technology is more flexible and convenient, with lower sensitivity to initial position errors and no accumulation of errors [16].

Geomagnetic matching technology involves matching the measured geomagnetic data with a pre-established geomagnetic database to find similar geomagnetic sequences, thereby determining the current position. The real-time capability and accuracy of the positioning are predominantly determined by the performance of the geomagnetic matching algorithm [17]. Traditional geomagnetic matching algorithms encompass the Iterative Closest Contour Point (ICCP) algorithm and the Geomagnetic Contour Matching (MAGCOM) algorithm [18]. The MAGCOM algorithm rectifies position errors in the INS through translational search, but when there is a large heading deviation, this algorithm cannot provide accurate positioning [19]. The ICCP algorithm can correct both heading and position errors of the INS. However, the ICCP algorithm typically suffers from significant initial localization errors and tends to fall into local optima [20]. To optimize the ICCP algorithm, Xiao et al. [21] introduced the Probability Data Association (PDA) algorithm and the principle of incremental modulation, which enhanced the algorithm’s robustness to interference and improved positioning accuracy.

In recent years, in addition to traditional geomagnetic matching algorithms, artificial intelligence algorithms and intelligent optimization algorithms have also been applied to geomagnetic matching navigation. Xu et al. [22] presented the PSO-ICCP algorithm, optimizing the ICCP output by incorporating a multi-attribute decision-making mechanism. By combining Particle Swarm Optimization (PSO) algorithm with an improved particle initialization strategy, this method effectively reduces the effect of initial positioning errors with respect to the geomagnetic matching precision of ICCP. Chen et al. [23] introduced the fundamentals of pattern recognition into geomagnetic matching navigation and proposed a geomagnetic vector matching algorithm based on a two-stage neural network. This method cascades the non-fully connected neural network and the probabilistic neural network (PNN) to perform preliminary and refined screening on the geomagnetic vectors and their characteristic information, respectively. This approach achieves a high matching success rate and positioning accuracy under low-gradient conditions, addressing the issue of failure in traditional geomagnetic matching algorithms under the same conditions. Similarly, inspired by the fundamentals of pattern recognition, they later proposed a geomagnetic vector matching method based on PNN. By optimizing the smoothing parameters of the PNN using a genetic algorithm, this method significantly improves matching accuracy compared to traditional geomagnetic matching algorithms [24].

Although existing geomagnetic matching algorithms achieve high matching accuracy, accurately and rapidly determining the position through geomagnetic information in the high-speed dynamic environment remains a challenge. In real-world environments, geomagnetic information is uniquely associated with positional information, which aligns with the characteristics of a regression prediction task. Therefore, machine learning methods can be employed to fit this model, resulting in a trained geomagnetic matching model. Once the airborne equipment acquires the geomagnetic information, it can quickly match and obtain the positional information through this model, enabling accurate and real-time positioning.

The Extreme Learning Machine (ELM) algorithm, known for its efficiency as a machine learning approach, simplifies the training process of traditional neural networks by randomly generating input layer weights and biases, thereby eliminating iterative optimization steps and significantly enhancing training speed [25]. Therefore, the ELM algorithm has a significant advantage in applications that require large-scale data processing and emphasize real-time performance [26], making it suitable for geomagnetic matching tasks. However, this algorithm has certain limitations. Due to the random initialization of the weights and biases of the neural network, the performance of the ELM algorithm may exhibit instability across different datasets, which can affect its generalizability [27,28]. To address these shortcomings, Huang et al. [29] proposed the Kernel Extreme Learning Machine (KELM) to better handle linearly non-separable samples and improve robustness. Gao et al. [30] further employed the improved Dung Beetle Optimization (IDBO) algorithm for the optimization of the regularization coefficient and kernel parameters in KELM, which allowed for the accurate identification of projectile aerodynamic parameters. Wang et al. [31] employed the adaptive neuron clipping algorithm and the PSO algorithm to improve the ELM network. They then applied the adaptive boosting (AdaBoost) algorithm to iteratively train a series of weak learners, ultimately combining them to form a strong learner for achieving high-precision prediction. More studies have also employed intelligent optimization algorithms to optimize the hyperparameters of machine learning models [32,33]. These works demonstrate that such hybrid models can achieve more stable and accurate performance in regression prediction tasks.

In order to achieve efficient and stable geomagnetic matching, this paper will use an improved NGO (INGO) algorithm to optimize the initial weights and biases of the ELM model, and propose the INGO-ELM model. To enhance the persuasiveness of this article, the IGRF-13 model will be used to simulate geomagnetic data and the performance of the presented geomagnetic matching algorithm will be evaluated. The innovations introduced in this paper are as follows:

1. To strengthen the optimization capability of the NGO algorithm, this paper proposes three improvement measures for the NGO algorithm.
2. This paper presents, for the first time, the INGO-ELM algorithm for geomagnetic matching-assisted navigation, where the INGO algorithm is used to optimize the initial weights and biases of the ELM, effectively enhancing the accuracy and stability of the ELM algorithm in geomagnetic matching positioning tasks.
3. The geomagnetic matching dataset is generated using the IGRF-13 model to validate the matching performance of the geomagnetic matching assisted navigation algorithm proposed in this paper, with noise added to the dataset to simulate the complexity of real-world environments. The simulation results demonstrate that the INGO-ELM algorithm presented in this paper exhibits superior robustness and is capable of accomplishing the positioning task in real time with high-accuracy matching performance, even in the presence of observational errors from the magnetic sensor.

The organization of this paper is as outlined below: The IGRF-13 model and the construction methodology of the geomagnetic matching dataset are introduced in Section 2. Section 3 discusses the three improvements made to the NGO algorithm and analyzes their effectiveness, which is followed by the introduction of the INGO-ELM model proposed in this paper. Section 4 presents a comparative analysis of the geomagnetic matching test results between the INGO-ELM model and four other models, further testing the geomagnetic matching performance of each model after the addition of noise. Section 5 presents the conclusions and outlook of this paper.

To ensure clarity and consistency in scientific communication, we have compiled a terminology list and an acronyms list, as shown in Table 1 and Table 2, respectively.

## 2. Dataset Construction and Data Preprocessing

The IGRF-13 (International Geomagnetic Reference Field, 13th Edition) is the internationally recognized global geomagnetic model, used to precisely describe the temporal variations and spatial distribution of the Earth’s magnetic field [34]. The model is based on integrated data from satellite observations, ground measurements, and airborne data. Through high-precision spherical harmonic expansion and numerical computation methods, it provides a detailed representation of the geomagnetic field from the ground to high altitudes. The IGRF-13 model covers a variety of geomagnetic field components, ranging from the Earth’s internal main field to external sources, effectively depicting the time-varying characteristics in the Earth’s magnetic field and contributing to the prediction of long-term magnetic field changes. This model finds extensive use in areas such as navigation, space weather forecasting, satellite orbit determination, and geophysical research, making it a critical tool in geomagnetic studies and applications. This paper constructs and preprocesses a dataset based on the IGRF-13 model for the purpose of training and testing geomagnetic matching algorithms.

### 2.1. Dataset Construction

The IGRF-13 model encompasses geomagnetic field data from 2020 to 2025, and the model demonstrates high accuracy across various altitudes, latitudes, longitudes, and time scales. In the IGRF-13 model, the spherical harmonic functions representing the geomagnetic potential are given by Equation (1):

(1) $$V=a\sum_{n=1}^{\infty }\sum_{m=0}^{n}{\left(\frac{a}{z}\right)}^{n+1}\left(h_{n}^{m}\sin m\lambda +g_{n}^{m}\cos m\lambda \right)P_{n}^{m}\left(\cos\theta \right)$$

In the equation, the Earth’s average radius is assumed as *a* = 6371.2 km; let *z* represent the distance from the Earth’s core to the calculation point, where z=a+h, with *h* being the distance from the Earth’s surface. The angle θ is the cotangent latitude, measured from the North Pole, and is given by $\theta =90^{\circ }-\varphi$, where φ is the geomagnetic latitude; the terms $h_{n}^{m}$ and $g_{n}^{m}$ are the normalized Schmidt spherical harmonic coefficients, also known as Gauss coefficients, with a cutoff degree of *n*; λ denotes the longitude, measured eastward from Greenwich. $P_{n}^{m}\left(\cos\theta \right)$ is the Schmidt quasi-normalized associated Legendre function of degree *n* and order *m*, defined as follows:

(2) $$\left\{\begin{array}{c} P_{n}^{m}\left(\cos\theta \right)=\frac{1}{2^{n}n!}\sqrt{\frac{C_{m}\left(n-m\right)!{\left(1-\cos^{2}\theta \right)}^{m}}{\left(n+m\right)!}}\frac{d^{n+m}{\left(\cos^{2}\theta -1\right)}^{n}}{d\cos\theta^{n+m}}\\ C_{m}=\left\{\begin{array}{c} 1\left(m=0\right)\\ 2\left(m\leq 1\right) \end{array}\right. \end{array}\right.$$

**Figure 1:**
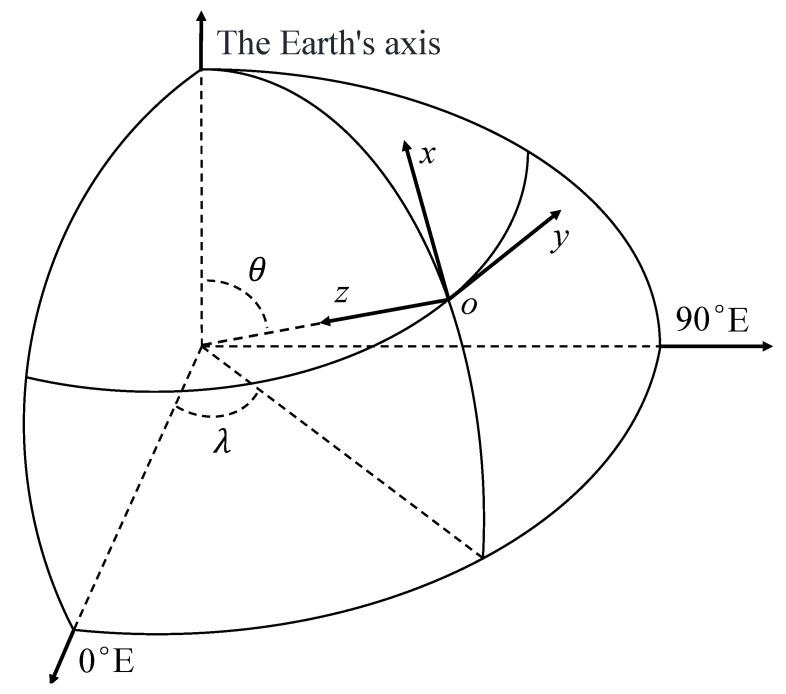
The North–East–Down coordinate system.

The coordinate system O-XYZ shown in Figure 1 represents the North–East–Down (NED) coordinate system when the Earth is modeled as a sphere. By applying the principle of potential field transformation, the magnetic potential *V* is differentiated with respect to the north, east, and vertical directions. This allows the components of the magnetic field strength in the three directions $B_{x}$, $B_{y}$, and $B_{z}$ to be obtained, as expressed by the following equations:

(3) $$\left\{\begin{array}{c} B_{x}=\frac{1}{z}\frac{\partial V}{\partial \theta }=\sum_{n=1}^{\infty }\sum_{m=0}^{n}{\left(\frac{a}{z}\right)}^{n+2}\left(h_{n}^{m}\sin m\lambda +g_{n}^{m}\cos m\lambda \right)\frac{d}{d\theta }P_{n}^{m}\left(\cos\theta \right)\\ B_{y}=\frac{1}{z\sin\theta }\frac{\partial V}{\partial \theta }=\sum_{n=1}^{\infty }\sum_{m=0}^{n}{\left(\frac{a}{z}\right)}^{n+2}\left(\frac{m}{\sin\theta }\right)\left(g_{n}^{m}\sin m\lambda -h_{n}^{m}\cos m\lambda \right)P_{n}^{m}\left(\cos\theta \right)\\ B_{z}=\frac{\partial V}{\partial z}=\sum_{n=1}^{\infty }\sum_{m=0}^{n}{\left(\frac{a}{z}\right)}^{n+2}\left(-n-1\right)\left(h_{n}^{m}\sin m\lambda +g_{n}^{m}\cos m\lambda \right)P_{n}^{m}\left(\cos\theta \right) \end{array}\right.$$

After obtaining the position information of a specific location, the components of the magnetic field strength along the three axes of the NED coordinate system at that location can be computed using Equation (1) through Equation (3). Therefore, this study first employs the IGRF-13 model to generate a dataset composed of coordinates, following the sampling approach outlined in Table 3.

After sampling, the geomagnetic information corresponding to all coordinates in the sampling area is calculated using the IGRF-13 model, resulting in a dataset composed of geomagnetic data. In the geomagnetic matching model proposed in this paper, the components of the total magnetic field intensity along the NED coordinate axes at the sampling points are used as input data, while the latitude, longitude, and altitude of the sampling points are the output data. These two datasets are paired one-to-one and will be allocated to the training, validation, and testing sets following an 8:1:1 proportion.

### 2.2. Data Preprocessing

In the computations based on the IGRF-13 model, the inputs are latitude, longitude, and altitude, while the outputs are the components of the total magnetic field strength along the three axes of the NED coordinate system. These features exhibit different scales and a wide range of values. Therefore, it is essential to normalize these features prior to subsequent model training. Data normalization is a widely used preprocessing technique, primarily aimed at transforming data with varying ranges or scales into a consistent scale, thereby preventing bias during model training due to discrepancies in feature scales. This research utilizes the min–max normalization method, which eliminates the scale differences between features by linearly transforming each feature value into the range of [0, 1]. The expression for this method is as follows:

(4) $$Y^{\prime }=\frac{Y-Y_{min}}{Y_{max}-Y_{min}}$$

In the equation, *Y* represents the original data; $Y_{max}$ and $Y_{min}$ represent the maximum and minimum values of this feature, respectively; and $Y^{\prime }$ represents the normalized data.

## 3. Geomagnetic Matching Algorithm

The aerial vehicle can obtain the components of the magnetic field intensity along the three axes of the NED coordinate system through the magnetic sensor. If these components can be rapidly matched to determine the position of the aerial vehicle, the positioning errors accumulated over time by the inertial navigation system can be corrected. Therefore, designing an efficient geomagnetic matching algorithm can enhance the accuracy, real-time capabilities, and stability of the aerial vehicle positioning. To achieve this objective, this paper proposes a geomagnetic matching algorithm obtained by integrating the Extreme Learning Machine (ELM) with the improved Northern Goshawk Optimization (INGO) algorithm.

### 3.1. Extreme Learning Machine

Extreme Learning Machine is a learning algorithm based on a single hidden layer feedforward neural network. Unlike traditional methods for training neural networks, the key advantage of ELM lies in its approach of randomly generating the connection weights and biases of the hidden layer nodes, and then computing the output layer weights using only the least-squares method. This significantly improves learning efficiency. The process does not require iterative optimization, thus avoiding the multiple computations and adjustments required by the backpropagation algorithm, greatly reducing training time and computational overhead. In regression and prediction tasks, ELM, through its randomized hidden layer structure, can map the input data to a high-dimensional feature space, effectively capturing the nonlinear relationships within the data. Since the training process does not involve iterative optimization, ELM not only exhibits high training efficiency but also mitigates the overfitting problems often encountered by traditional neural networks, offering more stable prediction performance. ELM is particularly well-suited for handling large-scale datasets, as it can significantly enhance training efficiency while maintaining high accuracy [35,36,37].

Given *N* distinct training samples, with the activation function denoted as $g\left(x\right)$, let the ELM network have *n* input neurons, *p* output neurons, and *l* hidden layer neurons. The ELM network can be mathematically represented as follows:

(5) Hβ=T

(6) $$H={\left[\begin{array}{ccc} g\left(w_{1}x_{1}+b_{1}\right) & \ldots & g\left(w_{l}x_{1}+b_{l}\right)\\ \vdots & \ddots & \vdots \\ g\left(w_{1}x_{N}+b_{1}\right) & \ldots & g\left(w_{l}x_{N}+b_{l}\right) \end{array}\right]}_{N\times l}$$

(7) $$W=\left[\begin{array}{c} \begin{array}{c} w_{1}\\ w_{2} \end{array}\\ \begin{array}{c} \vdots \\ w_{l} \end{array} \end{array}\right]={\left[\begin{array}{ccc} w_{11} & \ldots & w_{1n}\\ \vdots & \ddots & \vdots \\ w_{l1} & \ldots & w_{ln} \end{array}\right]}_{l\times n}$$

(8) $$\beta =\left[\begin{array}{c} \beta_{1}\\ \beta_{2}\\ \begin{array}{c} \vdots \\ \beta_{l} \end{array} \end{array}\right]={\left[\begin{array}{ccc} \beta_{11} & \ldots & \beta_{1n}\\ \vdots & \ddots & \vdots \\ \beta_{l1} & \ldots & \beta_{lm} \end{array}\right]}_{l\times m}$$

(9) $$b=\left[\begin{array}{c} b_{1}\\ b_{2}\\ \begin{array}{c} \vdots \\ b_{l} \end{array} \end{array}\right]$$

In the equations, *H* represents the output matrix of the hidden layer; *W* denotes the weight matrix between the input layer and the hidden layer; β represents the weight matrix between the output layer and the hidden layer; *T* represents the matrix of expected outputs for the training samples; and *b* refers to the threshold of the hidden layer. The parameters of the hidden layer neurons in *b* and *W* are randomly generated, and once the training samples are provided, the matrix *H* is known. To solve for the matrix β, Equation (5) can be transformed into solving the linear system Hβ=T, which can be obtained by the minimum norm least-squares solution $\hat{\beta }$:

(10) $$\hat{\beta }=H^{+}T$$

In the equation, $H^{+}$ represents the Moore–Penrose generalized inverse of the hidden layer output matrix *H*.

Despite the numerous advantages of ELM, its main drawback lies in the random initialization of the hidden layer weights, which may limit the model’s stability and prediction accuracy.

### 3.2. The Northern Goshawk Optimization Algorithm and Its Improvements

The Northern Goshawk Optimization (NGO) algorithm, introduced by Dehghani et al. [38], is a novel population-based optimization approach. Compared to other algorithms, the basic framework of this algorithm demonstrates better optimization capabilities; however, its search ability still has room for improvement. In this paper, three improvement measures for the NGO algorithm are proposed.

#### 3.2.1. The Northern Goshawk Optimization Algorithm

The mathematical model established based on the different hunting stages of the NGO algorithm is presented as follows.

Stage 1: Prey identification (exploration process).

Prey is freely distributed in the environment, and its behavior is modeled by the following expression:

(11) $$P_{i}=X_{c},\;i=1,2,\ldots ,N;\;c=1,2,\ldots ,N$$

$P_{i}$ represents the location of the target prey for the *i*-th Northern Goshawk; $X_{c}$ denotes the state of the Northern Goshawk; *c* is a natural number within the interval [1, *N*]; and *N* is the total population of goshawks.

A prey is randomly chosen by the Northern Goshawk, which then launches a swift attack. The behavioral model of the goshawk is expressed by Equations (12) and (13).

(12) $$X_{i,u}^{new,p1}=\left\{\begin{array}{c} x_{i,u}+s\left(p_{i,u}-Ix_{i,u}\right),{\;F}_{si}<F_{i}\\ x_{i,u}+s\left(x_{i,u}-p_{i,u}\right),{\;\;\;F}_{si}\geq F_{i} \end{array}\right.$$

(13) $$X_{i}=\left\{\begin{array}{c} X_{i}^{new,p1},F_{i}^{new,p1}<F_{i}\\ X_{i}\;\;\;\;\;\;\;\;,F_{i}^{new,p1}\geq F_{i} \end{array}\right.$$

In the equations, $F_{si}$ represents the ideal fitness value; $X_{i}^{new,p1}$ denotes the *i*-th Northern Goshawk’s new state; $X_{i,u}^{new,p1}$ represents the *i*-th Northern Goshawk’s new state in the *u*-th dimension; *s* is an arbitrary value within the range [1, *N*], used to generate the erratic behavior of the goshawk; *I* takes the values of 1 or 2; and $F_{i}^{new,p1}$ is the fitness value associated with the *i*-th Northern Goshawk’s new state in the *u*-th dimension.

Stage 2: Pursuit and evasion (development process).

During the hunting process of the Northern Goshawk, the prey attempts to escape. It is assumed that the Northern Goshawk pursues its prey within a range of radius $R_{1}$. The mathematical expression for this process is as follows:

(14) $$X_{i,u}^{new,p2}=x_{i,u}+R_{1}\left(2s-1\right)x_{i,u}$$

(15) $$R_{1}=0.02\left(1-\frac{j}{J}\right)$$

(16) $$X_{i}=\left\{\begin{array}{c} X_{i}^{new,p2},{\;F}_{i}^{new,p2}<F_{i}\\ X_{i\;\;\;\;},{\;F}_{i}^{new,p2}\geq F_{i} \end{array}\right.$$

In the equations, $F_{i}^{new,p2}$ represents the new fitness value under the new state; $X_{i,u}^{new,p2}$ represents the *i*-th Northern Goshawk’s new state in the *u*-th dimension during the second stage; *j* represents the current iteration number; *J* indicates the maximum iteration limit; and $X_{i}^{new,p2}$ represents the *i* -th Northern Goshawk’s new state during the second stage.

#### 3.2.2. Improvement Measures for the NGO Algorithm

The local development capability and global search ability of the NGO algorithm require further enhancement, and its stability, convergence speed, and accuracy are also insufficient. To address these issues, this paper proposes three improvement measures:

Improvement measure 1: Bernoulli shift mapping initialization strategy.

Enhancing the diversity of the initial population plays a key role in improving the global search capability of the algorithm, enabling it to explore a broader solution space and preventing the algorithm from prematurely becoming stuck in local optima. However, in the first stage of the NGO algorithm, the Northern Goshawks are randomly distributed in an uneven manner, resulting in uneven individual quality within the population and preventing the algorithm from obtaining the optimal initial positions. Therefore, this paper adopts the Bernoulli shift mapping initialization strategy [39] to optimize the NGO algorithm. The Bernoulli shift mapping, as an efficient nonlinear mapping, can be used to generate initialization populations with high randomness and chaotic characteristics. Its formula can be expressed as follows:

(17) $$x_{k+1}=\left\{\begin{array}{c} \frac{x_{k}}{1-\varepsilon },\;\;\;\;\;\;\;0<x_{k}\leq 1-\varepsilon \\ \frac{x_{k}-1+\varepsilon }{\varepsilon },\;\;1-\varepsilon <x_{k}<1 \end{array}\right.$$

In the equation, *k* denotes the number of mappings; ε is the adjustment coefficient, set to 0.4 in this paper; $x_{k}$ the sequence value generated by the *k*-th shift mapping; and $x_{0}$ denotes a random value in the interval (0, 1).

**Figure 2:**
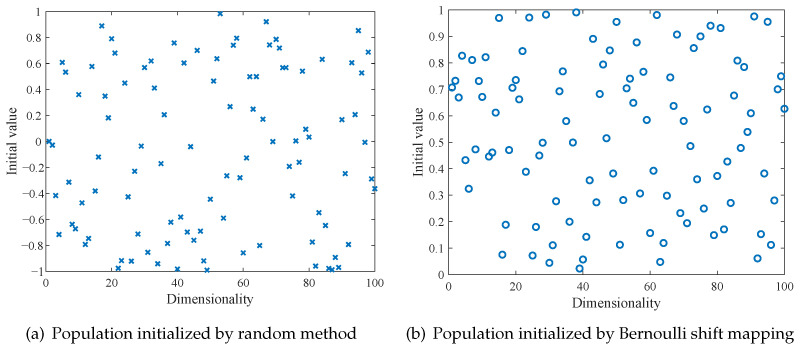
Comparison of initial population distributions.

Figure 2a,b illustrate the population distributions generated by the random method and the Bernoulli shift mapping, respectively. Comparatively, it is evident that the population generated by the Bernoulli shift mapping exhibits a more uniform distribution throughout the entire space, with fewer local clusters.

Improvement measure 2: Gaussian mutation strategy.

Since the NGO algorithm is prone to become stuck in local optima, the Gaussian mutation strategy is employed in this paper to optimize the algorithm. Gaussian mutation generates new solutions by introducing random perturbations to the individual’s state through random numbers drawn from a Gaussian distribution (normal distribution), thereby expanding the exploration range of the solution space, enhancing solution quality, and accelerating convergence [40]. Gaussian mutation provides strong support for solution diversity and global search, effectively improving the ability of the NGO algorithm to escape from local optimum. In the optimization process of the NGO algorithm, the Gaussian mutation strategy is applied when the fitness function value stays constant for five successive iterations. Its specific expression is as follows:

(18) $${X_{i}}^{\prime }=X_{i}\left(1+\gamma \right)$$

In the expression, γ is a random number that follows a Gaussian distribution, with a maximum value of 1 and a minimum value of 0; $X_{i}$ represents the current state of the individual; and ${X_{i}}^{\prime }$ denotes the state of the individual after applying Gaussian mutation.

Improvement measure 3: Improved nonlinear convergence factor.

In the second stage of the NGO algorithm, the goshawk pursues its prey within an attack range of radius $R_{1}$, which is related to the current iteration number *j* and the maximum iteration number *J*, and gradually reduces as the current iteration number grows. In the early stage of the goshawk’s hunting, the value of $R_{1}$ is relatively large. To enhance the global search capability of the NGO algorithm, the decay rate of $R_{1}$ should be slowed down during this phase to maintain a larger search step size, thereby avoiding local optima. In the later stage of the goshawk’s hunting, when $R_{1}$ becomes smaller and the search step size of the algorithm decreases, the decay rate of $R_{1}$ should be increased to ensure that it remains small in the latter stage of the optimization process, improving the accuracy of the algorithm’s search. However, as shown in Equation (15), $R_{1}$ changes linearly and does not adequately meet these requirements. Therefore, this paper improves the NGO algorithm by using a nonlinear convergence factor $R_{2}$, which is expressed as follows:

(19) $$R_{2}=0.01\left[\cos\left(\frac{\pi \cdot j}{J}\right)+1\right]$$

**Figure 3:**
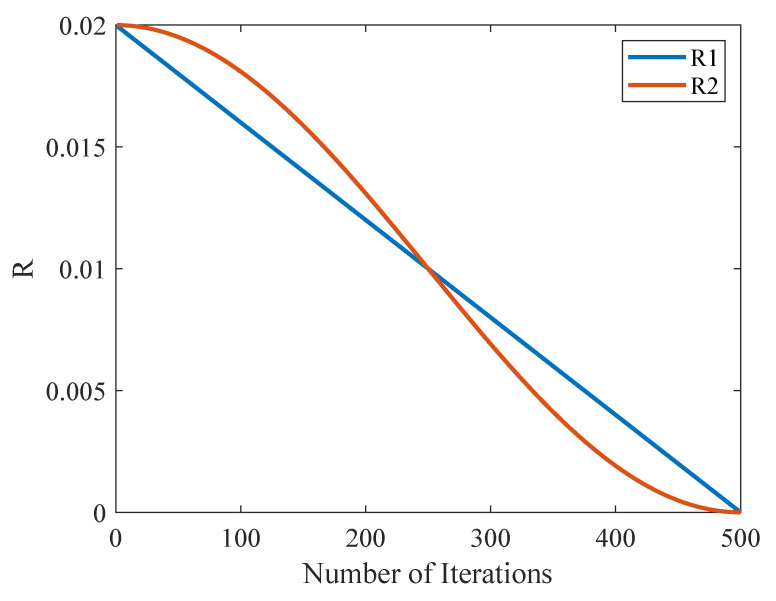
Comparison of convergence factors.

The original convergence factor $R_{1}$ and the improved convergence factor $R_{2}$ are shown in Figure 3, with their variations across iterations. $R_{2}$ decays more slowly than $R_{1}$ in the early stages of the algorithm’s optimization, while it decays more rapidly than $R_{1}$ in the later stage, which is consistent with the expected results.

#### 3.2.3. Analysis of the Effectiveness of the Improvement Measures

To verify the effectiveness of the three improvement measures in the NGO algorithm, performance tests were conducted on the improved algorithm, INGO. The results were compared with several state-of-the-art algorithms, including NGO, GWO [41], WOA [42], and PSO [43]. The parameter settings for these algorithms are provided in Table 4.

In this study, the CEC2005 benchmark function suite was used to assess the performance of these algorithms. As detailed in Table 5, the CEC2005 benchmark function suite includes 23 test functions. $F_{1}-F_{7}$ are unimodal test functions, each having a single global optimum within the given range, which are capable of testing the accuracy and convergence rate of the algorithms. $F_{8}-F_{13}$ are multimodal test functions, characterized by a single global optimum within the given interval, but multiple local optima. These functions are utilized to evaluate the algorithm’s global search capability and its ability in avoiding local optima. $F_{14}-F_{23}$ are fixed-dimensional multimodal test functions, with lower dimensions and fewer extrema, designed to simultaneously test the accuracy, convergence rate, and global search capability of the algorithm. The performance of different algorithms is compared based on the mean values and standard deviations across these three types of test functions. A lower average value signifies greater convergence accuracy of the algorithm, whereas a smaller standard deviation reflects better stability of the algorithm.

In this test, each algorithm was executed 30 times independently, with the number of iterations limited to 500 and a population size of 30. The test results are presented in Table 6.

Based on the test results of the unimodal test functions $F_{1}-F_{5}$, $F_{7}$, the multimodal test functions $F_{9}-F_{11}$, $F_{13}$, and the fixed-dimensional multimodal test functions $F_{14}-F_{20}$, $F_{23}$, it is evident that the INGO algorithm consistently achieves higher convergence accuracy than the other four algorithms, and in most cases, it also demonstrates superior stability. In the test results of other functions, the convergence accuracy of the INGO algorithm shows only a small difference compared to the best-performing algorithm, highlighting its excellent performance. Overall, the improvements proposed for the NGO algorithm in this paper effectively enhance the optimization capability and stability of the algorithm.

**Figure 4:**
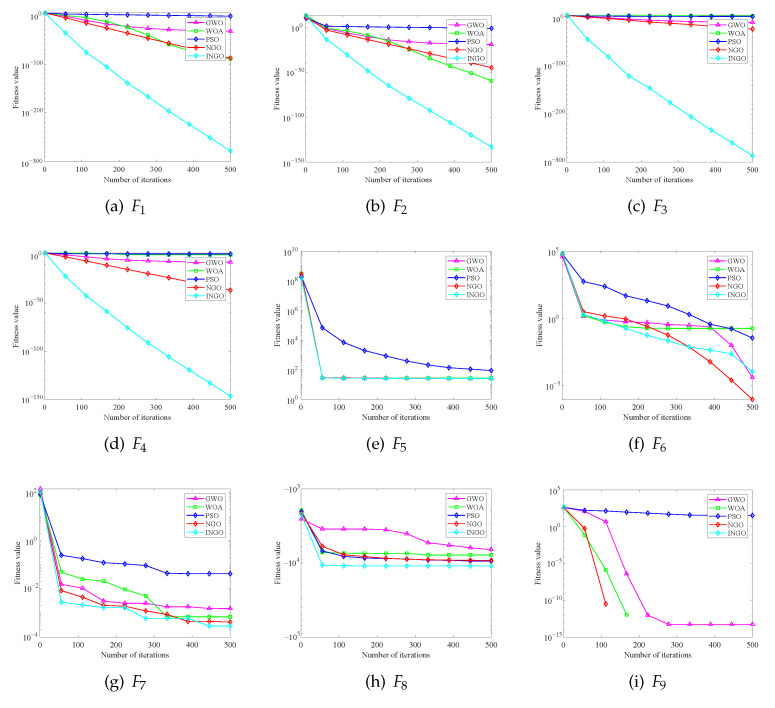

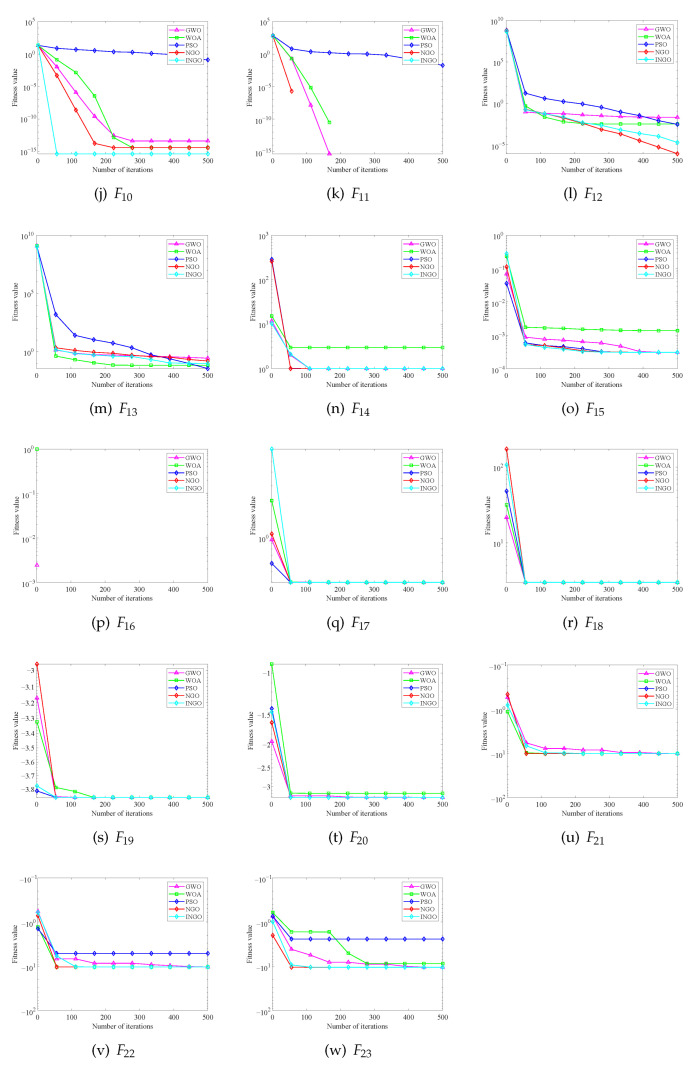
Convergence curves of various intelligent optimization algorithms on the CEC2005 benchmark function suite.

To provide a more intuitive comparison of the convergence and optimization processes between the INGO algorithm and other algorithms, Figure 4 illustrates the convergence curves of five algorithms on 23 benchmark test functions. The dimensions of functions $F_{1}-F_{13}$ are 30, where the vertical axis corresponds to the fitness function values, and the horizontal axis corresponds to the iteration counts of the algorithms. As shown in Figure 4, although the INGO algorithm performs poorly in the convergence curves of the fitness functions for $F_{6}$ and $F_{12}$, it exhibits the best convergence speed and precision among the five algorithms for other benchmark functions. In most cases, the INGO algorithm achieves better convergence accuracy with fewer iterations and demonstrates a powerful capability to avoid local optima. In particular, in the convergence curves of $F_{1}-F_{4}$, the global optimization ability of the INGO algorithm exhibits global optimization capability that significantly outperforms other algorithms.

In conclusion, based on the test results of the five algorithms, the INGO algorithm shows significant advantages in terms of convergence rate, accuracy, and stability, and stability, thereby validating the effectiveness of the improvement measures proposed to the NGO algorithm in this paper.

### 3.3. INGO-ELM Geomagnetic Matching Algorithm

**Figure 5:**
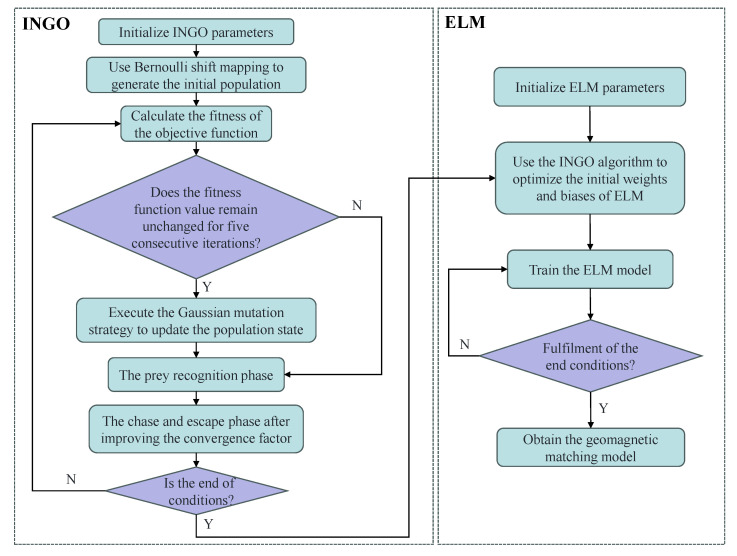
Flowchart of the INGO-ELM algorithm.

Despite the fast training speed and high prediction accuracy of ELM, the random initialization of the hidden layer weights and biases can lead to instability in the model’s predictive performance. This issue is particularly evident when the training data contains noise or outliers, which may compromise the accuracy and robustness of the model. To optimize the ELM algorithm, this paper utilizes the INGO algorithm to initialize the hidden layer weights and biases of the ELM model, thereby proposing the INGO-ELM geomagnetic matching algorithm. Figure 5 presents the flowchart of the algorithm.

Based on Figure 5, the INGO-ELM model is trained on the training set constructed in Section 2, resulting in the geomagnetic matching model.

## 4. Simulation Tests

The INGO-ELM geomagnetic matching model introduces the principle of machine learning to form a one-to-one nonlinear mapping between geomagnetic information and position information for direct matching and positioning. By leveraging the advantages of machine learning, this method can not only perform matching based on existing geomagnetic databases but also accurately estimates the geomagnetic data between discrete points in the database, essentially constructing a continuous and complete geomagnetic matching database to achieve more precise matching and positioning.

Thus, to verify the geomagnetic matching performance of the INGO-ELM model, we construct geomagnetic matching models using XGBoost and BP neural networks—both machine learning algorithms—and compared them with the INGO-ELM model in simulation tests. To ensure a fair comparison comparison with the INGO-ELM model, we use INGO to optimize the parameters of XGBoost and BP neural networks. Meanwhile, we built two geomagnetic matching models, NGO-ELM and ELM, to further validate the performance of INGO as an intelligent optimization algorithm.

### 4.1. Extreme Gradient Boosting

Extreme gradient boosting (XGBoost) [44], a classic algorithm in the field of machine learning, originates from the optimization and upgrading of the gradient boosting decision tree (GBDT) in its core design philosophy. When training on large-scale datasets, XGBoost significantly accelerates the training speed through efficient tree structure pruning and parallel computing mechanisms. Meanwhile, it introduces regularization terms to prevent overfitting, enabling the model to maintain excellent generalization performance on complex datasets. As the core of XGBoost, its objective function Obj formula is as follows:

(20) $$Obj=\sum_{i=1}^{n}L\left(y_{i},{\hat{y}}_{i}\right)+\Omega \left(\hat{f}\right)$$

$L\left(y_{i},\hat{y_{i}}\right)$ denotes the loss function, which is employed to measure the discrepancy between the model’s predicted value $\hat{y_{i}}$ and the true value $y_{i}$. $\Omega \left(\hat{f}\right)$ represents the regularization term, serving to control the complexity of each tree. Its formula is

(21) $$\Omega \left(\hat{f}\right)=\gamma_{1}T_{1}+\frac{1}{2}\lambda_{1}\sum_{j=1}^{m}w_{j}^{2}$$

In the formula, $\hat{f}$ is the prediction function; $\gamma_{1}$ is the regularization parameter; $T_{1}$ is the number of leaf nodes in the regression tree; $\lambda_{1}$ is the penalty term for leaf node weights; *m* is the number of features; and $w_{j}$ is the leaf node weight of the *j*-th tree.

### 4.2. Back Propagation Neural Network

The Back Propagation (BP) Neural Network [45], a feedforward neural network, exhibits a strong nonlinear fitting capability and finds extensive applications. When constructing a geomagnetic matching model using a BP neural network in this paper, the input layer consists of three nodes, which are the components of the total magnetic field intensity at the sampling point along the three axes of the NED coordinate system. The hidden layer is set with 10 nodes. The output layer contains three nodes, corresponding to the latitude, longitude, and altitude of the sampling point, respectively. The activation functions of neurons in the hidden layer and output layer are shown in Equations (22) and (23), respectively:

(22) $$f_{1}\left(x\right)=\frac{e^{x}-e^{-x}}{e^{x}+e^{-x}}$$

(23) $$f_{2}\left(x\right)=x$$

### 4.3. Geomagnetic Matching Simulation Test

To establish the control group, INGO is used to optimize the number of iterations, tree depth, and learning rate of XGBoost. Similarly, INGO is employed to optimize the weight matrix from the input layer to the hidden layer, the weight matrix from the hidden layer to the output layer, and the biases of the hidden layer and output layer for the BP neural network.

Then, the geomagnetic matching dataset constructed in Section 2 is used to conduct comparative tests on five models, namely, INGO-ELM, NGO-ELM, ELM, INGO-XGBoost, and INGO-BP. The test results are shown in Table 7, Table 8 and Table 9, which present the error statistics of latitude, longitude, and height obtained by the five geomagnetic matching models, respectively.

In Table 7, Table 8 and Table 9, the INGO-ELM model exhibits the lowest mean absolute error (MAE), root mean square error (RMSE), and mean square error (MSE) among the five models, indicating that the average errors for latitude, longitude, and altitude obtained through this model are the smallest, with the fewest prediction outliers. Therefore, the geomagnetic matching accuracy and stability of this model are the highest. Furthermore, taking the MAE evaluation index as an example, the MAE for latitude and longitude obtained using the INGO-ELM geomagnetic matching model are 5.728 × 10^−5^ degrees and 5.98 × 10^−5^ degrees, respectively, which correspond to approximately 6.38 m and 6.43 m after unit conversion. The MAE for altitude obtained by this model is 0.0137 m. These results demonstrate that the geomagnetic matching model based on INGO-ELM achieves extremely high matching accuracy.

The error evaluation index of the NGO-ELM model are second only to those of the INGO-ELM model, remaining within the same order of magnitude. However, it is noteworthy that in the latitude matching results presented in Table 7, the maximum error of the INGO-ELM model is 6.156 × 10^−4^ degrees, which corresponds to approximately 68.53 m after unit conversion, while the maximum error of the NGO-ELM model is 9.554 × 10^−4^ degrees, equivalent to approximately 106.36 m after unit conversion, with a difference of about 37.83 m between the two. Similarly, in the longitude matching results shown in Table 8, the maximum error of the two models differs by approximately 41.74 m after unit conversion. A positioning error of several tens of meters has a significant impact on the navigation of the aerial vehicle. Therefore, compared to the NGO-ELM algorithm, the INGO-ELM algorithm not only enhances the matching accuracy but also greatly improves stability. The improvements proposed for the NGO algorithm in this paper are effective and have practical application value.

From Table 7, Table 8 and Table 9, it can be observed that the four error evaluation index for latitude and altitude obtained through the INGO-BP model are similar to those of the INGO-ELM model. However, for longitude, the MAE value of the BP neural network model is 8.359 × 10^−4^, which differs by an order of magnitude from the MAE value of 5.98 × 10^−5^ for the INGO-ELM model, demonstrating relatively poor performance. This discrepancy may be attributed to the tendency of the BP neural network to become stuck in local optima during the training process, leading to lower overall geomagnetic matching accuracy. The error data of the INGO-XGBoost model in the three tables are mostly two orders of magnitude higher than those of the INGO-ELM model. This could be attributed to the fact that the matching results of XGBoost are determined by the expected values based on the conditional distribution of features, lacking the ability to excavate deeper-level relationships between features. XGBoost cannot directly capture the complex feature relationships in the geomagnetic matching dataset, resulting in the poor geomagnetic matching performance of INGO-XGBoost model. In Table 7 and Table 8, all error evaluation metrics for the ELM model are one order of magnitude higher than those of the INGO-ELM and NGO-ELM models, indicating that optimizing the initial weights and biases of the ELM model using the INGO or NGO algorithms significantly improves its matching performance.

Furthermore, in the geomagnetic matching dataset of this paper, the units of latitude and longitude are degrees, with small numerical values and minimal variations, while the unit of altitude is meters, featuring large numerical values and significant variations. However, the data obtained by the machine learning model is dimensionless, and the model itself does not inherently understand the specific physical meanings of each input and output parameter. Without considering dimensional units, the output data in Table 7, Table 8 and Table 9 show that the matching errors for latitude and longitude are several orders of magnitude lower than those for altitude. However, after converting the units of latitude and longitude from degrees to meters, their matching errors become higher than those of altitude. This indicates that the dimensional discrepancies in the geomagnetic matching dataset lead to higher positioning accuracy for altitude.

**Figure 6:**
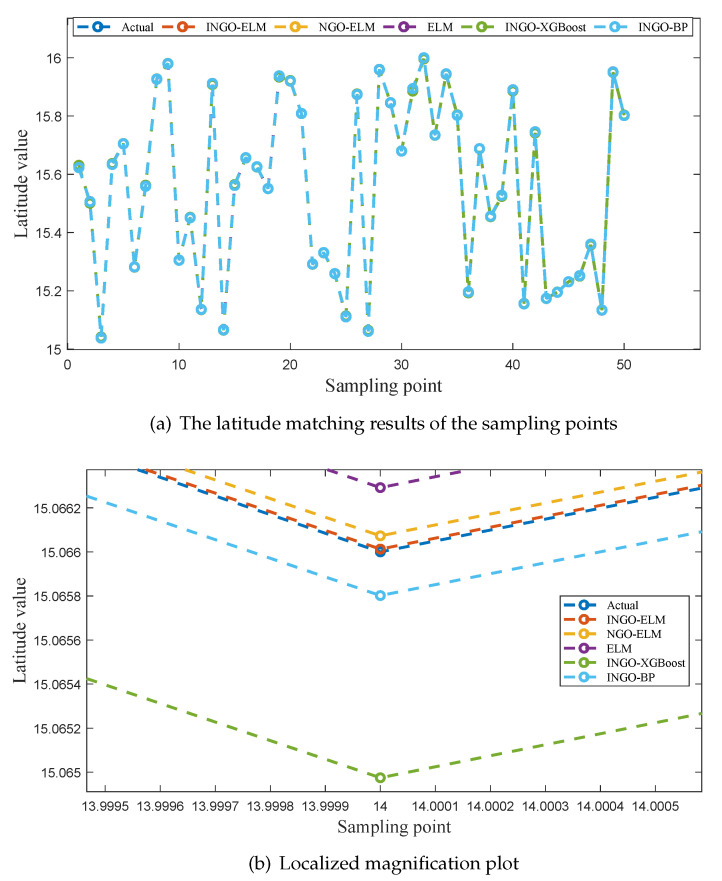
The latitude matching results of different models.

**Figure 7:**
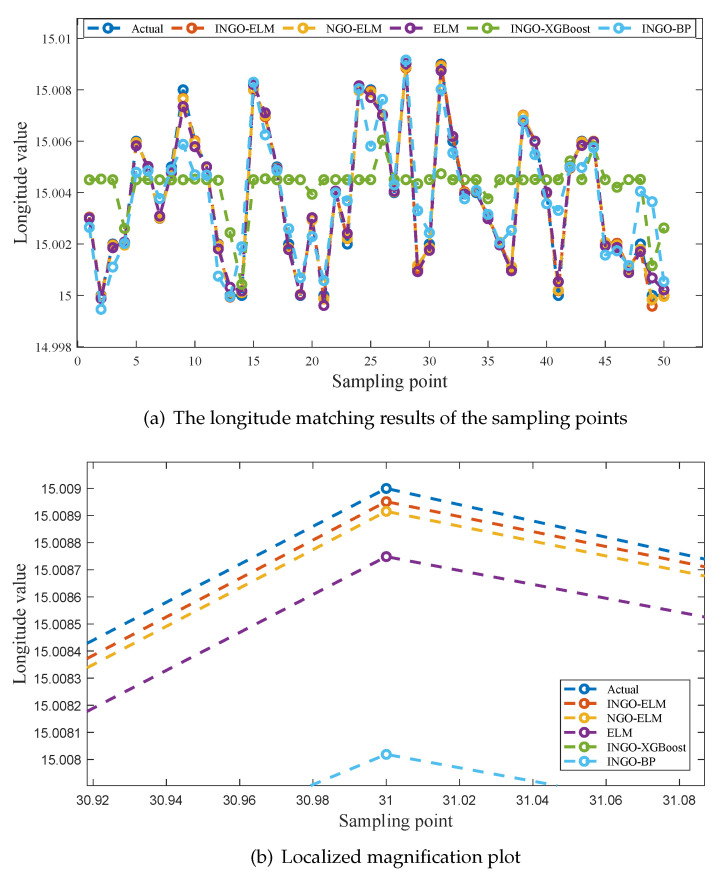
The longitude matching results of different models.

**Figure 8:**
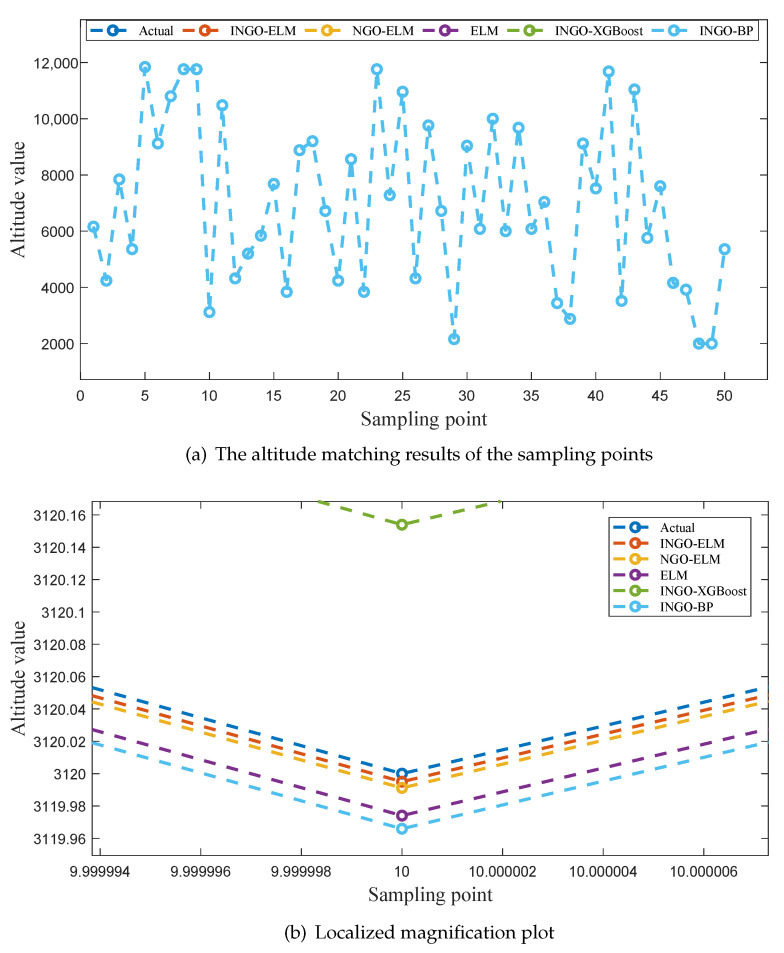
The altitude matching results of different models.

To provide a more intuitive comparison of the fitting performance among the five models, Figure 6a, Figure 7a and Figure 8a illustrate the predicted values of latitude, longitude, and altitude for the sampling points by different models, along with the corresponding true values. Enlarged sections of these three figures are shown in Figure 6b, Figure 7b, and Figure 8b.

As seen in Figure 6a and Figure 8a, the predicted values of five models exhibit a good fit with the actual values at all sampling points. However, in Figure 7a, it is evident that INGO-XGBoost and INGO-BP models exhibit poor fitting performance, while the other three algorithms still perform well. This situation may be attributed to the fact that within the sampling range of this study, the variation in longitude [15, 15.009] is much smaller than the variation in latitude [15, 16], while the sampling interval is the same for both at 0.001 degrees. As a result, the longitude sampling values lack sufficient diversity, and both INGO-XGBoost and INGO-BP models failed to capture key features when processing this type of data, leading to poor prediction performance. As shown in Figure 6b, Figure 7b, and Figure 8b, the INGO-ELM model demonstrates the highest matching accuracy.

Based on the comprehensive analysis of Table 7, Table 8 and Table 9 and Figure 6, Figure 7, and Figure 8, the test results confirm the effectiveness of the three improvement measures proposed for NGO in this study. In addition, after obtaining the geomagnetic data of the aerial vehicle’s location, the trained INGO-ELM model can accurately determine the aerial vehicle’s geographic coordinates in just 0.27 µs. Therefore, the proposed INGO-ELM geomagnetic matching model significantly outperforms NGO-ELM, ELM, INGO-XGBoost and INGO-BP models, achieving stable, precise, and real-time positioning.

It is worth noting that achieving the positioning accuracy of the INGO-ELM geomagnetic matching model imposes stringent requirements on geomagnetic sensors. Taking latitude as an example for analysis, in the IGRF-13 model, when longitude and altitude remain unchanged, the geomagnetic field may change by only a few nanoteslas (nT) per 0.001 degrees of latitude (approximately 110 m). In the simulation results of this paper, the average absolute error in latitude matching is 6.38 m, implying that to achieve this positioning accuracy, the sensitivity of the geomagnetic sensor must be lower than 1 nT. However, as shown in References [46,47,48,49], with the development of atomic magnetometer technology, geomagnetic sensors will be capable of achieving picotesla (pT)-level or even femtotesla (fT)-level three-axis magnetic field measurements within a high dynamic range in noisy and physically demanding environments, meeting the performance requirements of the geomagnetic matching method proposed in this paper.

### 4.4. Robustness Verification

Within the sampling range shown in Table 3, the interference of non-natural noise on the magnetic field is minimal. This study primarily focuses on the impact of observational errors from the geomagnetic sensor on the INGO-ELM geomagnetic matching model. To evaluate the robustness of the INGO-ELM geomagnetic matching model, Gaussian white noise with standard deviations of 1 nT and 5 nT was added to the input data of the test set, and the test results are presented in Table 10, Table 11 and Table 12.

In Table 10 and Table 11, the INGO-ELM model exhibits the smallest mean absolute error for geomagnetic matching, indicating that the model maintains the highest geomagnetic matching accuracy among the five models even after the introduction of Gaussian white noise. Furthermore, these two tables demonstrate that after adding noise, the geomagnetic matching mean absolute error of INGO-ELM and NGO-ELM is significantly better than that of the other three models. When the standard deviation of the Gaussian white noise is 1nT, the matching accuracy of the two models is similar. However, when the standard deviation of the Gaussian white noise is 5 nT, the mean absolute error of the INGO-ELM model in matching latitude and longitude, after unit conversion, is approximately 12.41 m and 12.28 m lower than that of the NGO-ELM model, respectively. These test results suggest that the INGO-ELM model demonstrates significantly better anti-interference capability than the NGO-ELM model.

Table 12 further indicates that when Gaussian white noise with a standard deviation of 1nT is added to the INGO-ELM geomagnetic matching model, the order of magnitude in most error data remains unchanged. However, when the standard deviation increases to 5 nT, the average absolute errors of the INGO-ELM model in matching latitude, longitude, and altitude, after unit conversion, become 15.83 m, 17.55 m, and 0.027 m, respectively, still maintaining a high level of geomagnetic matching accuracy. Meanwhile, the maximum errors, after unit conversion, become 213.63 m, 196.13 m, and 0.409 m, but their occurrence probability is extremely low, and abnormal values can be eliminated using filtering algorithms. Therefore, a comprehensive analysis of the data in Table 8, Table 9 and Table 10 reveals that the INGO-ELM model not only demonstrates excellent matching accuracy but also maintains robust performance in the presence of noise.

## 5. Conclusions

Geomagnetic matching-assisted navigation offers several advantages, including all-terrain, all-weather capabilities, strong anti-jamming performance, and no error accumulation, which can compensate for the shortcomings of the INS/GPS integrated navigation system. The INGO-ELM geomagnetic matching model proposed in this paper enables real-time, precise, and stable positioning for high-speed aerial vehicles. In this model, an improved NGO algorithm is used to optimize the initial weights and biases of the ELM model, and the effectiveness of the improvements is validated using the CEC2005 benchmark function suite. The results demonstrate that the INGO algorithm performs best overall in the tests, and the improvements effectively enhance the convergence rate, convergence accuracy, and stability of the NGO algorithm.

In addition, this paper employs the IGRF-13 model to generate a geomagnetic matching dataset and conducts comparative testing of several geomagnetic matching models, including INGO-ELM, NGO-ELM, ELM, INGO-XGBoost, and INGO-BP. The simulation results show that after the airborne equipment acquires the geomagnetic data, it only takes 0.27 µs to obtain the latitude, longitude, and altitude of the aerial vehicle through the INGO-ELM model. After unit conversion, the average absolute errors are approximately 6.38 m, 6.43 m, and 0.0137 m, respectively. When considering all types of geomagnetic matching error data comprehensively, the geomagnetic matching performance of the INGO-ELM model is significantly superior to that of the other four models. Furthermore, the INGO-ELM model maintained a high level of geomagnetic matching accuracy even after the addition of Gaussian white noise to the test set inputs, indicating that the model exhibits good robustness. Therefore, the INGO-ELM algorithm presented in this paper exhibits superior robustness and is capable of accomplishing the positioning task in real time with high-accuracy matching performance, even in the presence of observational errors from the magnetic sensor.

Future Outlook: Although the INGO-ELM model demonstrates exceptionally high geomagnetic matching accuracy, it requires precise geomagnetic data from the target area for training. A major challenge lies in the stable acquisition of such data in advance. Although the IGRF-13 model adopted in this study is a highly internationally recognized geomagnetic field reference model, it exhibits certain discrepancies compared to the real geomagnetic field. In fact, the geomagnetic matching algorithm proposed herein is equally applicable to datasets generated by other more accurate geomagnetic field models that establish one-to-one correspondences between geomagnetic information and location information. In future work, we will focus on the construction of regional geomagnetic field models. Additionally, geomagnetic matching-assisted navigation may be severely disrupted in special circumstances, such as geomagnetic storms. Therefore, it is crucial to explore how to integrate the INGO-ELM model with other navigation systems to effectively address such situations.

## Figures and Tables

**Table 1:** Terminology summary.

Mathematical Symbol	Full Name
*V*	Geomagnetic potential
*a*	The Earth’s average radius
*z*	The distance from the Earth’s core to the calculation point
θ	Cotangent latitude measured from the North Pole
φ	Geomagnetic latitude
λ	Longitude measured eastward from Greenwich
$g_{n}^{m},h_{n}^{m}$	Normalized Schmidt spherical harmonic coefficients
$P_{n}^{m}\left(\cos\theta \right)$	Schmidt quasi-normalized associated Legendre function of degree *n* and order *m*
$B_{x},B_{y},B_{z}$	Magnetic field intensities in northward, eastward, and downward directions
*H*	Output matrix of the hidden layer in ELM
$H^{+}$	Moore–Penrose generalized inverse of H
*W*	Weight matrix between the input layer and the hidden layer in ELM
β	Weight matrix between the output layer and the hidden layer in ELM
*T*	Expected output matrix of training samples in ELM
*b*	Threshold of the hidden layer in ELM
$\hat{\beta }$	Minimum norm least squares solution
$P_{i}$	Location of the target prey for the *i*-th Northern Goshawk
$X_{c}$	State of the Northern Goshawk
$F_{si}$	Ideal fitness value of NGO algorithm
$X_{i}^{new,p1}$	New state of the *i* -th Northern Goshawk at Stage 1 of NGO algorithm
$X_{i,u}^{new,p1}$	New state of the *i* -th Northern Goshawk in the u-th dimension at Stage 1 of NGO algorithm
$F_{i}^{new,p1}$	Fitness value of Northern Goshawk under the new state at Stage 1 of NGO algorithm
$X_{i}^{new,p2}$	New state of the *i* -th Northern Goshawk at Stage 2 of NGO algorithm
$X_{i,u}^{new,p2}$	New state of the *i* -th Northern Goshawk in the u-th dimension at Stage 2 of NGO algorithm
$F_{i}^{new,p2}$	Fitness value of Northern Goshawk under the new state at Stage 2 of NGO algorithm
$R_{1}$	Convergence factor of stage 2 in NGO algorithm
$R_{2}$	Convergence factor of stage 2 in INGO algorithm
*J*	Maximum iteration limit of stage 2 in NGO or INGO algorithm
ε	Adjustment coefficient of the Bernoulli shift map
γ	A random number that follows a Gaussian distribution
$a_{1}$	Linear decreasing convergence factor in GWO algorithm
$b_{1}$	Spiral shape parameter in WOA
$c_{1},\;c_{2},\;w_{1}$	Cognitive parameter, social parameter, and inertia weight in PSO algorithm
Obj	Objective function in XGBoost
$\hat{f}$	Prediction function in XGBoost
$\gamma_{1}$	Regularization parameter in XGBoost
$T_{1}$	Number of leaf nodes in the regression tree of XGBoost
$\lambda_{1}$	Penalty term for leaf node weights in XGBoost
$\hat{y_{i}}$	Model predicted values in XGBoost
$y_{i}$	True value
$L\left(y_{i},\hat{y_{i}}\right)$	Loss function in XGBoost
$\Omega \left(\hat{f}\right)$	Regularization term in XGBoost
$f_{1}\left(x\right)$	Activation functions of neurons in the hidden layers of BP neural network
$f_{2}\left(x\right)$	Activation functions of neurons in the output layer of BP neural network

**Table 2:** Acronyms summary.

Acronyms	Full Name
NGO	Northern Goshawk Optimization
INGO	Improved Northern Goshawk Optimization
ELM	Extreme Learning Machine
XGBoost	Extreme Gradient Boosting
BP	Back Propagation
PSO	Particle Swarm Optimization
GWO	Grey Wolf Optimizer
WOA	Whale Optimization Algorithm
INS	Inertial Navigation System
GPS	Global Positioning System
IGRF-13	International Geomagnetic Reference Field, 13th Edition
SITAN	Sandia Inertial Terrain Aided Navigation
ICCP	Iterative Closest Contour Point
MAGCOM	Geomagnetic Contour Matching
PDA	Probability Data Association
PNN	Probabilistic Neural Network
KELM	Kernel Extreme Learning Machine
IDBO	Improved Dung Beetle Optimization
AdaBoost	Adaptive Boosting
NED	North–East–Down (coordinates system)
CEC	Congress on Evolutionary Computation
GBDT	Gradient Boosting Decision Tree

**Table 3:** The sampling approach for latitude, longitude, and altitude.

Sampling Parameters	Sampling Range	Sampling Interval
Latitude (°)	[15, 16]	0.001
Longitude (°)	[15, 15.009]	0.001
Altitude (m)	[2000, 12,000]	80

**Table 4:** Parameter settings of the Intelligent Optimization Algorithm.

Algorithm	Name of the Parameter	Value of the Parameter
INGO	$R_{2}$	[0, 0.02]
NGO	$R_{1}$	[0, 0.02]
GWO	$a_{1}$	[0, 2]
WOA	$b_{1}$	1
PSO	$c_{1}$, $c_{2}$, $w_{1}$	1.49618, 1.49618, 0.8

**Table 5:** The CEC2005 benchmark function suite.

Function Expressions	Dimension	Range	Optimal Value
$F_{1}\left(x\right)=\sum_{i=1}^{d}x_{i}^{2}$	30/100	[−100, 100]	0
$F_{2}\left(x\right)=\sum_{i=1}^{d}\left|x_{i}\right|+\prod_{i=1}^{d}\left|x_{i}\right|$	30/100	[−100, 100]	0
$F_{3}\left(x\right)=\sum_{i=1}^{d}{\left(\sum_{j=1}^{i}x_{j}\right)}^{2}$	30/100	[−10, 10]	0
$F_{4}\left(x\right)=\max\{\left|x_{i}\right|,\;1\leq i\leq d\}$	30/100	[−100, 100]	0
$F_{5}\left(x\right)=\sum_{i=1}^{d-1}\left.\left[{\left(x_{i}-\;1\right)}^{2}+100{\left(x_{i+1}-x_{i}^{2}\right)}^{2}\right.\right]$	30/100	[−30, 30]	0
$F_{6}\left(x\right)=\sum_{i=1}^{d}{\left(\left[x_{i}+0.5\right]\right)}^{2}$	30/100	[−100, 100]	0
$F_{7}\left(x\right)=random\left[0,\;1\right)+\sum_{i=1}^{d}ix_{i}^{4}$	30/100	[−1.28, 1.28]	0
$F_{8}\left(x\right)=\sum_{i=1}^{d}-x_{i}sin\left(\sqrt{\left|x_{i}\right|}\right)$	30/100	[−500, 500]	−418.982× dim
$F_{9}\left(x\right)=\sum_{i=1}^{d}\left.\left[x_{i}^{2}-10cos\left(2\pi x_{i}\right)+10\right.\right]$	30/100	[−5.12, 5.12]	0
$F_{10}\left(x\right)=-exp\left.\left(\frac{1}{d}\sum_{i=1}^{d}cos\left(2\pi x_{i}\right)\right.\right)-20exp\left.\left(-0.2\sqrt{\frac{1}{d}\sum_{i=1}^{d}x_{i}^{2}}\right.\right)+e+20$	30/100	[−32, 32]	0
$F_{11}\left(x\right)=1+\prod_{i=1}^{d}cos\left.\left(\frac{x_{i}}{\sqrt{i}}\right.\right)+\frac{1}{4000}\sum_{i=1}^{d}x_{i}^{2}$	30/100	[−600, 600]	0
$F_{12}\left(x\right)=\frac{\pi }{d}\left\{{\left(y_{n}-1\right)}^{2}+\sum_{i=1}^{d-1}\left.\left[10sin^{2}\left(\pi y_{i+1}\right)+1\right.\right]{\left(y_{i}-1\right)}^{2}\right.$	30/100	[−50, 50]	0
$\;\;\;\;\;\;\;\;\;\;\;\left.+10sin\left(\pi y_{1}\right)\right\}+\sum_{i=1}^{d}u\left(x_{i},10,100,4\right)$			
$\;\;\;\;\;\;\;y_{i}=\frac{1+x_{i}}{4}+1$			
$\;\;\;\;\;\;\;u\left(x_{i},a,k,m\right)=\left\{\begin{array}{c} k{\left(-a+x_{i}\right)}^{m},x_{i}>a\\ 0,-a<x_{i}<a\\ k{\left(-a-x_{i}\right)}^{m},x_{i}<-a \end{array}\right.$			
$F_{13}\left(x\right)=\sum_{i=1}^{d}u\left(x_{i},5,100,4\right)+0.1\left.\{{\left(x_{d}-1\right)}^{2}\left.\left[sin^{2}\left(2\pi x_{d}\right)+1\right.\right]\right.$	30/100	[−50, 50]	0
$\;\;\;\;\;\;\;\;\;\;\left.+\sum_{i=1}^{d}{\left(1+x_{i}\;\right)}^{2}\left.\left[sin^{2}\right.\left(1+3\pi x_{i}\;\right)+1\right]+sin^{2}\left(3\pi x_{i}\right)\right\}$			
$\;\;\;\;\;\;\;\;u\left(x_{i},a,k,m\right)=\left\{\begin{array}{c} k{\left(-a+x_{i}\right)}^{m},x_{i}>a\\ 0,-a<x_{i}<a\\ k{\left(-a-x_{i}\right)}^{m},x_{i}<-a \end{array}\right.$			
$F_{14}\left(x\right)={\left.\left(\frac{1}{500}+\sum_{j=1}^{25}\frac{1}{i+\sum_{i=1}^{2}x_{i}-a_{ij}}\right.\right)}^{-1}$	2	[−65.53, 65.53]	1
$F_{15}\left(x\right)=\sum_{i=1}^{11}{\left.\left[a_{i}-\frac{x_{i}\left(b_{i}^{2}+\;b_{i}x_{2}\right)}{b_{i}^{2}+\;b_{i}x_{3}+\;x_{4}}\right.\right]}^{2}$	4	[−5, 5]	0.0003075
$F_{16}\left(x\right)=x_{1}x_{2}+\frac{1}{3}x_{1}^{6}-2.1x_{1}^{4}+4x_{1}^{2}+4x_{2}^{4}-4x_{2}^{2}$	2	[−5, 5]	−1.0316285
$F_{17}\left(x\right)={\left.\left(x_{2}-\frac{5.1}{4\pi^{2}}x_{1}^{2}+\frac{5}{\pi }x_{1}-6\right.\right)}^{2}+10\left.\left(1-\frac{1}{8\pi }\right.\right)cos\;x_{1}+10$	2	[−5, 10]× [0, 15]	0.398
$F_{18}\left(x\right)=\left.\left[\left(3x_{1}^{3}-14x_{1}+3x_{2}^{2}+6x_{1}x_{2}-14x_{2}+19\right){\left(x_{2}+x_{1}+1\right)}^{2}+1\right.\right]$	2	[−5, 5]	3
$\;\;\;\times \left.\left[\left(12x_{1}^{2}-32x_{1}+27x_{2}^{2}-36x_{1}x_{2}+48x_{2}+18\right)\times {\left(2x_{1}-3x_{2}\right)}^{2}+30\right.\right]$			
$F_{19}\left(x\right)=-\sum_{i=1}^{4}c_{1}exp\left(-\sum_{j=1}^{3}a_{ij}{\left(-p_{ij}+x_{j}\right)}^{2}\right)$	3	[0, 1]	−3.86
$F_{20}\left(x\right)=-\sum_{i=1}^{4}c_{1}exp\left(-\sum_{j=1}^{6}a_{ij}{\left(-p_{ij}+x_{j}\right)}^{2}\right)$	6	[0, 1]	−3.32
$F_{21}\left(x\right)=-\sum_{i=1}^{5}{\left.\left[c_{i}+\left(-a_{i}+X\right){\left(-a_{i}+X\right)}^{T}\right.\right]}^{-1}$	4	[0, 10]	−10.1523
$F_{22}\left(x\right)=-\sum_{i=1}^{7}{\left.\left[c_{i}+\left(-a_{i}+X\right){\left(-a_{i}+X\right)}^{T}\right.\right]}^{-1}$	4	[0, 10]	−10.4028
$F_{23}\left(x\right)=-\sum_{i=1}^{10}{\left.\left[c_{i}+\left(-a_{i}+X\right){\left(-a_{i}+X\right)}^{T}\right.\right]}^{-1}$	4	[0, 10]	−10.5363

**Table 6:** The test results of various intelligent optimization algorithms on the CEC2005 benchmark function suite.

Function	Item	INGO	NGO	GWO	WOA	PSO	Rank
$F_{1}$	AVG	$3.15\times 10^{-262}$	$1.75\times 10^{-88}$	$6\times 10^{-33}$	$2.76\times 10^{-85}$	0.063392	1
	STD	0	$3.77\times 10^{-88}$	$1.34\times 10^{-32}$	$9\times 10^{-85}$	0.073493	1
$F_{2}$	AVG	$3\times 10^{-130}$	$9.80\times 10^{-46}$	$8.5\times 10^{-20}$	$4.33\times 10^{-54}$	0.369031	1
	STD	$1.1\times 10^{-129}$	$7.37\times 10^{-46}$	$7.67\times 10^{-20}$	$2.14\times 10^{-53}$	1.823812	1
$F_{3}$	AVG	$5.2\times 10^{-266}$	$5.59\times 10^{-23}$	$1.68\times 10^{-8}$	29,390.27	1304.786	1
	STD	0	$2.37\times 10^{-22}$	$3.38\times 10^{-8}$	11,871.37	1100.218	1
$F_{4}$	AVG	$1.3\times 10^{-131}$	$8.34\times 10^{-38}$	$2.07\times 10^{-8}$	40.84204	5.535247	1
	STD	$7.2\times 10^{-131}$	$6.36\times 10^{-38}$	$2.23\times 10^{-8}$	29.04829	1.084562	1
$F_{5}$	AVG	25.07017	25.48175	26.73398	27.43498	3147.53	1
	STD	0.197656	0.378691	0.666209	0.381211	16412.4	1
$F_{6}$	AVG	0.007508	$9.45\times 10^{-7}$	0.524527	0.111634	0.090861	2
	STD	0.038813	$4.3\times 10^{-7}$	0.354699	0.112065	0.082604	2
$F_{7}$	AVG	0.000264	0.000497	0.00125	0.001825	0.030162	1
	STD	0.000139	0.000238	0.000657	0.001988	0.010857	1
$F_{8}$	AVG	−7641.21	−6759.21	−6090.95	−10843.1	−8131.35	3
	STD	343.3791	569.1969	896.4646	1636.906	689.8551	1
$F_{9}$	AVG	0	0	1.680008	0	43.41142	1
	STD	0	0	2.829896	0	13.16153	1
$F_{10}$	AVG	$4.44\times 10^{-16}$	$5.3\times 10^{-15}$	$4.31\times 10^{-14}$	$4.23\times 10^{-15}$	0.162205	1
	STD	0	$1.74\times 10^{-15}$	$5.36\times 10^{-15}$	$1.85\times 10^{-15}$	0.255072	1
$F_{11}$	AVG	0	0	0.000588	0	0.152681	1
	STD	0	0	0.002236	0	0.120431	1
$F_{12}$	AVG	$1.19\times 10^{-5}$	$2.68\times 10^{-7}$	0.031227	0.006675	0.151436	2
	STD	$5.33\times 10^{-6}$	$3.08\times 10^{-7}$	0.019466	0.006805	0.293799	2
$F_{13}$	AVG	0.023122	0.06525	0.335469	0.201898	0.093056	1
	STD	0.029073	0.090489	0.230345	0.154582	0.079277	1
$F_{14}$	AVG	0.998004	0.998004	4.296612	1.786602	0.998004	1
	STD	0	0	4.047256	1.87817	$1.17\times 10^{-16}$	1
$F_{15}$	AVG	0.000308	0.000308	0.003776	0.000696	0.001147	1
	STD	$3.12\times 10^{-7}$	$8.79\times 10^{-7}$	0.007551	0.000383	0.003647	1
$F_{16}$	AVG	−1.03163	−1.03163	−1.03163	−1.03163	−1.03163	1
	STD	$6.78\times 10^{-16}$	$6.71\times 10^{-16}$	$1.88\times 10^{-8}$	$2.13\times 10^{-10}$	$6.32\times 10^{-16}$	3
$F_{17}$	AVG	0.397887	0.397887	0.397905	0.397892	0.397887	1
	STD	0	0	$8.56\times 10^{-5}$	$1.38\times 10^{-5}$	0	1
$F_{18}$	AVG	3	3	3.000014	3.000003	3	1
	STD	$9.4\times 10^{-16}$	$9.26\times 10^{-16}$	$1.68\times 10^{-5}$	$5.65\times 10^{-6}$	$1.07\times 10^{-15}$	2
$F_{19}$	AVG	−3.86278	−3.86278	−3.86154	−3.85961	3.86278	1
	STD	$2.71\times 10^{-15}$	$2.7\times 10^{-15}$	0.002591	0.002958	$2.64\times 10^{-15}$	3
$F_{20}$	AVG	−3.322	−3.322	−3.24887	−3.23504	−3.26037	1
	STD	$1.41\times 10^{-15}$	$2.95\times 10^{-14}$	0.07886	0.112313	0.063773	1
$F_{21}$	AVG	−10.1521	−10.1532	−9.30821	−9.05871	−6.30225	2
	STD	0.00399	$1.87\times 10^{-9}$	1.918351	2.531618	3.132277	2
$F_{22}$	AVG	−10.2257	−10.4029	−10.226	−9.14426	−8.5522	3
	STD	0.970414	$2.7\times 10^{-9}$	0.963122	2.521123	3.164457	3
$F_{23}$	AVG	−10.5364	−10.5364	−10.535	−8.65884	−7.92306	1
	STD	0.0001	$1.32\times 10^{-15}$	0.00079	2.769705	3.538868	2

**Table 7:** The latitude matching error (°) of different models.

Evaluation Index	INGO-ELM	NGO-ELM	ELM	INGO-XGBoost	INGO-BP
MAE	$5.728\times 10^{-5}$	$6.516\times 10^{-5}$	$1.139\times 10^{-4}$	$1.697\times 10^{-3}$	$8.284\times 10^{-5}$
MSE	$5.754\times 10^{-9}$	$8.013\times 10^{-9}$	$2.316\times 10^{-8}$	$5.664\times 10^{-6}$	$1.17\times 10^{-8}$
MAX	$6.156\times 10^{-4}$	$9.554\times 10^{-4}$	$1.016\times 10^{-3}$	$3.519\times 10^{-2}$	$1.119\times 10^{-3}$
RMSE	$7.586\times 10^{-5}$	$8.951\times 10^{-5}$	$1.522\times 10^{-4}$	$2.38\times 10^{-3}$	$1.082\times 10^{-4}$

**Table 8:** The longitude matching error (°) of different models.

Evaluation Index	INGO-ELM	NGO-ELM	ELM	INGO-XGBoost	INGO-BP
MAE	$5.98\times 10^{-5}$	$6.761\times 10^{-5}$	$1.289\times 10^{-4}$	$2.234\times 10^{-3}$	$8.359\times 10^{-4}$
MSE	$6.609\times 10^{-9}$	$8.399\times 10^{-9}$	$3.086\times 10^{-8}$	$7.058\times 10^{-6}$	$1.29\times 10^{-6}$
MAX	$5.305\times 10^{-4}$	$9.187\times 10^{-4}$	$1.434\times 10^{-3}$	$5.893\times 10^{-3}$	$4.984\times 10^{-3}$
RMSE	$8.13\times 10^{-5}$	$9.165\times 10^{-5}$	$1.757\times 10^{-4}$	$2.657\times 10^{-3}$	$1.136\times 10^{-3}$

**Table 9:** The altitude matching error (m) of different models.

Evaluation Index	INGO-ELM	NGO-ELM	ELM	INGO-XGBoost	INGO-BP
MAE	$1.372\times 10^{-2}$	$1.635\times 10^{-2}$	$3.028\times 10^{-2}$	0.104	$5.11\times 10^{-2}$
MSE	$2.82\times 10^{-4}$	$4.467\times 10^{-4}$	$1.495\times 10^{-3}$	$3.16\times 10^{-2}$	$4.698\times 10^{-3}$
MAX	0.104	0.202	0.324	19.099	0.314
RMSE	$1.68\times 10^{-2}$	$2.114\times 10^{-2}$	$3.867\times 10^{-2}$	0.178	$6.854\times 10^{-2}$

**Table 10:** Mean absolute errors under Gaussian white noise with standard deviation of 1 nT.

Coordinate Parameters	INGO-ELM	NGO-ELM	ELM	INGO-XGBoost	INGO-BP
Latitude (°)	$7.818\times 10^{-5}$	$8.583\times 10^{-5}$	$4.691\times 10^{-4}$	$2.128\times 10^{-3}$	$2.949\times 10^{-4}$
Longitude (°)	$9.416\times 10^{-5}$	$9.919\times 10^{-5}$	$6.763\times 10^{-4}$	$2.615\times 10^{-3}$	$9.091\times 10^{-4}$
Altitude (m)	$1.522\times 10^{-2}$	$1.827\times 10^{-2}$	$7.95\times 10^{-2}$	0.162	0.159

**Table 11:** Mean absolute errors under Gaussian white noise with standard deviation of 5 nT.

Coordinate Parameters	INGO-ELM	NGO-ELM	ELM	INGO-XGBoost	INGO-BP
Latitude (°)	$1.422\times 10^{-4}$	$2.537\times 10^{-4}$	$7.557\times 10^{-4}$	$3.952\times 10^{-3}$	$6.889\times 10^{-4}$
Longitude (°)	$1.632\times 10^{-4}$	$2.774\times 10^{-4}$	$1.105\times 10^{-3}$	$3.498\times 10^{-3}$	$1.263\times 10^{-3}$
Altitude (m)	$2.736\times 10^{-2}$	$4.078\times 10^{-2}$	0.123	0.568	0.372

**Table 12:** Matching errors of the INGO-ELM model under Gaussian white noise with different standard deviations.

Coordinate Parameters	0 nT		1 nT		5 nT	
	MAE	MAX	MAE	MAX	MAE	MAX
Latitude (°)	$5.728\times 10^{-5}$	$6.156\times 10^{-4}$	$7.818\times 10^{-5}$	$1.11\times 10^{-3}$	$1.422\times 10^{-4}$	$1.919\times 10^{-3}$
Longitude (°)	$5.98\times 10^{-5}$	$5.305\times 10^{-4}$	$9.416\times 10^{-5}$	$9.799\times 10^{-4}$	$1.632\times 10^{-4}$	$1.824\times 10^{-3}$
Altitude (m)	$1.372\times 10^{-2}$	0.104	$1.522\times 10^{-2}$	0.171	$2.736\times 10^{-2}$	0.409

## Data Availability

Data is contained within the article.
